# Treating tobacco dependence in older adults: a survey of primary care clinicians’ knowledge, attitudes, and practice

**DOI:** 10.1186/s12875-015-0317-7

**Published:** 2015-08-06

**Authors:** Lisa Huddlestone, Gemma Michelle Walker, Robana Hussain-Mills, Elena Ratschen

**Affiliations:** Division of Epidemiology & Public Health, The University Of Nottingham, Clinical Sciences Building, City Hospital Campus, Nottingham, NG5 1PB UK; Department of Medicine, CLAHRC EM, Institute of Mental Health, University of Nottingham, Nottingham, UK; NHS Nottingham Clinical Commissioning Group, Nottingham, UK

## Abstract

**Background:**

The benefits of smoking cessation among older people are well documented. Despite this, evidence suggests that older smokers are rarely engaged in smoking cessation efforts, and that existing tobacco dependence treatments require further tailoring to the specific needs of older smokers. This study assesses the knowledge, attitudes, and clinical practice of primary care clinicians in relation to addressing tobacco dependence among older people.

**Methods:**

A cross-sectional survey of 427 NHS primary care clinicians in a large English city was conducted using modified version of a previously validated questionnaire.

**Results:**

One hundred and seventy one clinicians (40 % response rate) completed the survey. While the majority (90.0 %) of respondents reported enquiring regularly about older patients’ smoking status, just over half (59.1 %) reported providing older patients with smoking cessation support. A lack of awareness in relation to the prevalence and impact of smoking in later life were apparent: e.g. only 47 % of respondents were aware of that approximately 10 life years are lost due to smoking related disease, and only 59 % knew that smoking can reduce the effectiveness of medication prescribed for conditions common in later life. Self-reported attendance at smoking-related training was significantly associated with proactive clinical practice.

**Conclusions:**

There is a need to improve clinicians’ knowledge, in relation to smoking and smoking cessation in older patients and to build clinician confidence in seizing teachable moments.

**Electronic supplementary material:**

The online version of this article (doi:10.1186/s12875-015-0317-7) contains supplementary material, which is available to authorized users.

## Background

While smoking prevalence among adults over 65 is lower (~10 %) than that of the younger population [1], the consequences of smoking later in life is substantial [2–6]. The smoking habits and quit motivations of older adults have been shown to differ substantially from those of younger smokers, being intrinsically connected to specific life experiences, strongly mediated by the idiosyncratic socio-cultural context in which they live [7–9]. Despite this, older smokers have been shown to be more likely to want to make a quit attempt, less likely to relapse than younger people, and to experience significant health benefits following cessation [10, 11].

With older people accounting for the highest activity and spend generated across primary care in England, having more regular and frequent contacts than the younger population [11], greater opportunities for the delivery of smoking cessation advice and treatment as recommended by the National Institute of Clinical Excellence (NICE) [12] exist. Despite this, and the proven effectiveness of smoking cessation treatment in older adults [13], many health professionals often fail to address tobacco dependence in older people [11, 14].

To our knowledge, no research has been conducted to explore issues relating to smoking and its treatment among older people in the context of primary care in England. This study explores primary care clinicians’ knowledge and attitudes in relation to tobacco use and its treatment among older people, as well as the clinical practices of identifying, advising, and treating older smokers in England.

## Methods

### Participants and setting

All (*n* = 427) health professionals working within 58 General practices (GP) and four specialist nursing teams (a Specialist Diabetes Nursing Service; a Community Matron Service; an Integrated Respiratory Service; and a Primary Care Cardiac Service) in a large city in the United Kingdom (UK) were invited to participate in the survey. Since 2013, all GP practices in England are accountable to local Clinical Commissioning Groups (CCGs), which also commission other health services, such as urgent and emergency care, elective hospital care, and rehabilitation services. Potential participants received an invitation to complete an online survey via email. While not incentivised, survey completion was encouraged by email cascade from practice and service managers two weeks after the initial invitation. Owing to a moderate uptake of the on-line survey, a paper version including return envelope was made available to all clinicians at their workplace, and was distributed with the assistance of a CCG Service Manager. Informed consent was obtained from all participants.

### Study instrument

The survey (Additional file 1) was developed from an instrument used in a previous study [15] and the findings of published research [3–6, 10, 16]. Twenty-nine closed and multiple choice questions, including Likert scales explored primary care professionals’ knowledge, attitudes, and current clinical practice related to tobacco dependence and its treatment among older people; additional questions related to reasons for not providing smoking cessation advice or treatment. An assessment of participants’ knowledge was made by asking respondents to select the correct answer from a choice of five possible answers to questions relating to the prevalence of smoking within both the general population in England, the prevalence of smoking in people over 65 years, and the number of life years lost to smoking related disease; an option of “don’t know” was included. Clinicians were asked to indicate their response of “correct” to a series of eight statements related to the impacts of smoking and quitting smoking in later life. Each of the statements provided was based upon the findings of previously published research [3–6, 10, 16]. Four false options (e.g. older people are less likely to want to quit smoking than younger smokers) were included.

Clinician attitudes towards addressing tobacco dependence among their older patients were assessed by asking respondents to rate their agreement with a series of statements on a 5-point Likert scale (strongly agree to strongly disagree). An exploration of clinician’s professional practice relating to the identification and treatment of older smokers was carried out using six questions which focused on self-reported practice in the previous month (e.g. How often did you provide older smokers with brief advice or encouragement to consider stopping smoking?). Respondents were asked to indicate from four options (always, often, rarely, or never) how often they undertook the six activities. Those clinicians who responded “rarely” or “never” were asked to provide additional information as to the reason for their selection from multiple choice options (e.g. ‘A number of the older people I have contact with have a terminal illness and I do not feel it would be appropriate to broach the subject of stopping smoking in these circumstances’.)

### Procedures

Responses were coded and entered in to IBM SPSS (version 22) and missing data from respondents were coded as such. Staff groups were categorised into “medical” (GPs, GP trainees, consultant physicians, and junior doctors) and “non-medical” (practice nurses, nurse prescribers, specialist nurses, community nurses, and healthcare assistants (HCA’s)). Where significant differences were detected between these groups, the non-medical staff group was sub-divided into qualified non-medical staff (nurses), and non-qualified non-medical staff (healthcare assistants) for more detailed analysis. Other a priori categories for analysis were based upon reported smoking status (current smoker v’s non-smoker), age range, gender, and reported attendance at training related to smoking.

In relation to participant knowledge of prevalence and the impact of smoking in later life on life years lost, responses were coded as correct or incorrect. To assess clinician knowledge in relation to the impact of smoking in older age, responses were coded and scored (1 = correct and 0 = incorrect) and an overall median score with the percentage correct answers was calculated from a maximum of 8. Attitudinal Likert scale responses were dichotomised for analysis, with “strongly agree” and “agree” forming one category and “neither agree nor disagree”, “disagree”, or “strongly disagree” forming the other. Responses to statements relating to professional practice were scored according to the procedure described by Kerr and colleagues [17] and an overall score was calculated from a maximum score of 18 points; responses to the statements relating to professional practice were dichotomised with “always” and “often” forming one category and “rarely” or “never” forming the second category.

### Data analysis

Means, medians, standard deviations, and proportions were obtained using descriptive analysis. Categorical data were analysed using Chi-squared tests to detect differences in outcome between the sub-groups; one-way ANOVA was used for continuous data; and logistic regression analysis was employed where statistical significance was indicated between subgroups to adjust for various significant factors; results are reported with odds ratios (OR) and 95 % confidence intervals (95 % CI). Statistical significance was taken to be *p* = 0.05.

### Ethical approval

Favourable ethical opinion was obtained from the University of Nottingham Faculty of Medicine and Health Sciences Research Ethics Committee (H13032014 SoM EPH 14026) and NHS research governance approval was granted by the local Clinical Commissioning Group (CCG) and the commissioned community health provider.

## Results

One hundred and seventy one clinicians responded to the survey (response rate = 40 %); 91 (21.3 %) completed the survey online and 80 (18.7 %) responded to the paper version.

### Participant characteristics

Responses were received from 83 (48.5 %) medical staff, 74 (43.3 %) qualified non-medical staff, 14 (8.2 %) non-qualified, non-medical staff. Over three quarters (76.6 %) of respondents were female; the overall median age range was 50–59 years; and 11 (6.4 %) respondents identified themselves as current smokers. Nearly two thirds (62.4 %) reported receiving smoking related training; 70.2 % of nurses reported training attendance compared to 58.5 % of physicians and 42.8 % of HCA’s. Further details of respondent characteristics can be found in Table 1. Differences in responses were not associated with gender, smoking status, or age range.

**Table 1:** Characteristics of survey respondents

Respondent characteristic		*n* = (%)
Gender	Male	40 (23.4)
	Female	131 (76.6)
Professional Role	GP	73 (42.7)
	Consultant Physician	4 (2.3)
	Junior Doctor	2 (1.2)
	Trainee GP	4 (2.4)
	Nurse Practitioner/Practice Nurse	55 (32.2)
	Community Nurse/Specialist Nurse	19 (11.1)
	Healthcare Assistant	14 (8.2)
Age Range	20-29 years	7 (4.1)
	30-39 years	39 (22.8)
	40-49 years	55 (32.2)
	50-59 years	61 (35.7)
	60-69 years	9 (5.3)
Smoking status	Current smoker	11 (6.4)
	Non-smoker	160 (93.6)
Attendance at smoking related training	Training	106 (62.4)
	No Training	64 (37.6)

### Knowledge of tobacco dependence and its treatment in older people

Significant differences were found between the knowledge of medical staff and non-medical staff. Table 2 provides details of the respondent knowledge of smoking, smoking cessation, and the impact of smoking and quitting in older age.

**Table 2:** Respondent knowledge of smoking, smoking cessation and the impact of smoking and smoking cessation in older age

Statement		All responses *n* = (%)	Professional Group [*n* = (%)]				Training Attendance [*n* = (%)]			
			Medical	Non-medical	Statistic *X* ^2^ (df = 1)	Sig p=	Training	No Training	Statistic *X* ^2^ (df = 1)	Sig p=
Approximately 20 % of the UK adult population smokes. (True)	Correct	79 (46.1)	43 (54.4)	36 (39.1)	4.00	0.048	48 (45.3)	31 (48.4)	0.16	0.752
	Incorrect	92 (53.9)	36 (45.6)	56 (60.9)			58 (54.7)	33 (51.6)		
Approximately 10 % of adults over the age of 65 smokes. (True)	Correct	23 (13.5)	10 (12.7)	13 (14.1)	0.07	0.825	11 (10.4)	12 (18.7)	2.39	0.164
	Incorrect	148 (86.5)	69 (87.3)	79 (85.9)			95 (89.6)	52 (81.3		
Approximately 10 life years are lost on average to smoking related disease. (True)	Correct	80 (46.8)	51 (64.5)	29 (31.5)	18.63	<0.001	48 (45.3)	31 (48.4)	0.16	0.752
	Incorrect	91 (53.2)	28 (35.5)	63 (68.5)			58 (54.7)	33 (51.6)		
Smoking can reduce the effectiveness of certain medications that are prescribed for conditions which are common in later life. (True)	Correct	101 (59.1)	51 (64.5)	50 (54.3)	1.83	0.213	63 (59.4)	37 (57.8)	0.04	0.873
	Incorrect	70 (40.1)	28 (35.5)	42 (45.7)			43 (40.6)	27 (42.2)		
Smoking can delay wound healing in older people. (True)	Correct	153 (89.4)	73 (92.4)	80 (86.6)	1.34	0.320	94 (88.7)	58 (90.6)	0.16	0.800
	Incorrect	18 (10.6)	6 (7.6)	12 (13.4)			12 (11.3)	6 (9.4)		
The risk of an older person having a heart attack or stroke increases within 24 h of stopping smoking. (False)	Correct	148 (86.5)	73 (92.4)	75 (81.5)	4.33	0.044	92 (86.7)	55 (85.9)	0.02	1.000
	Incorrect	23 (13.5)	6 (7.6)	17 (18.5)			14 (13.3)	9 (14.1)		
Smoking can speed cognitive decline in older people. (True)	Correct	129 (75.4)	75 (94.9)	54 (58.7)	30.13	<0.001	77 (72.6)	51 (79.7)	1.06	0.361
	Incorrect	42 (24.6)	4 (5.1)	38 (41.3)			29 (27.4)	13 (20.3)		
Smoking does not generally stop the progression of chronic obstructive pulmonary disease (COPD). (False)	Correct	135 (78.9)	67 (84.8)	68 (73.9)	3.08	0.093	89 (83.9)	45 (70.3)	4.45	0.052
	Incorrect	36 (21.1)	12 (15.2)	24 (26.1)			17 (16.1)	19 (29.7)		
Smoking can cause serious complications in older people with diabetes. (True)	Correct	162 (94.7)	78 (98.7)	84 (91.3)	4.71	0.030	99 (93.4)	62 (96.9)	0.96	0.486
	Incorrect	9 (5.3)	1 (1.3)	8 (8.7)			7 (6.6)	2 (3.1)		
Older smokers are less likely to want to quit smoking than younger smokers. (False)	Correct	93(54.4)	51 (64.5)	42 (45.6)	6.12	0.014	62 (58.5)	31 (48.4)	1.63	0.202
	Incorrect	78 (45.6)	28 (35.5)	50 (54.4)			44 (41.5)	33 (51.6)		
Brief smoking cessation advice is less effective than more intensive advice in helping older people to stop smoking. (False)	Correct	133 (77.8)	62 (79.7)	70 (76.1)	0.33	0.586	84 (79.2)	49 (76.6)	0.17	0.704
	Incorrect	38 (22.2)	16 (20.3)	22 (23.9)			22 (20.8)	15 (23.4)		

The median number of correct answers provided by respondents was 6 out of a maximum of 8 (75.0 %). Doctors achieved a mean score of 6.7 (SD 0.9); nurses a mean score of 5.8 (SD 1.4); and HCA’s a mean score of 4.7 (SD 1.1). Attendance at smoking related training was not found to be significantly associated with clinician knowledge of tobacco dependence and its treatment.

### Clinician attitudes regarding tobacco dependence treatment among older people

Overall, clinicians reported positive attitudes towards addressing tobacco dependence among older patients. However, in relation to perceptions of knowledge and skills in relation to identifying and treating tobacco dependence significantly more medical staff reported having this capacity than their non-medical colleagues.

Training attendance was found to be significantly associated with one attitudinal statement. Respondents who reported having attended training, were significantly less likely to hold the belief that “smoking is one of the few pleasures older people have” than those who had not attended training (81.0 % vs 67.2 %, *p =* 0.043). Table 3 shows the frequency of clinician attitudes towards addressing tobacco dependence among older patients by professional group and attendance at training on the attitudes of clinicians with regard to tobacco dependence and its treatment in older people.

**Table 3:** Frequency of clinician attitudes towards addressing tobacco dependence among older patients

Statement		All responses *n* = (%)	Professional Group *n* = (%)				Training Attendance *n* = (%)			
			Medical	Non-medical	Statistic *X* ^2^ (df=1)	Sig p=	Training	No Training	Statistic *X* ^2^ (df = 1)	Sig p=
I feel it lies within my remit of responsibility as a health professional to assist older people who smoke to address their tobacco dependence.	Agree	154 (90.0)	75 (94.9)	79 (85.9)	3.9	0.071	98 (92.4)	55 (85.9)	1.9	0.193
	Disagree	17 (10.0)	4 (5.1)	13 (14.1)			8 (7.6)	9 (14.1)		
I feel I have sufficient knowledge of the detrimental effects of smoking in later life to be able to discuss this effectively when I have contact with older patients who smoke.	Agree	134 (78.9)	69 (87.3)	65 (70.6)	6.9	0.009	86 (81.1)	47 (73.4)	1.4	0.255
	Disagree	37 (21.1)	10 (12.7)	27 (29.4)			20 (18.9)	17 (26.6)		
I feel I have sufficient knowledge of the benefits of stopping smoking in later life to be able to discuss this effectively with older patients who smoke.	Agree	135 (78.3)	71 (89.8)	64 (69.5)	10.5	0.001	87 (82.1)	47 (73.4)	1.8	0.245
	Disagree	36 (21.7)	8 (10.2)	28 (30.5)			19 (17.9)	17 (26.6)		
I feel I have sufficient knowledge of the barriers that may prevent older people from attempting to stop smoking.	Agree	102 (59.6)	48 (60.7)	54 (58.7)	0.1	0.876	69 (65.1)	32 (50.0)	3.8	0.056
	Disagree	69 (40.4)	31 (39.3)	38 (41.3)			37 (34.9)	32 (50.0)		
I feel I have sufficient knowledge of nicotine addiction/withdrawal to be able to discuss this effectively with older patients who smoke.	Agree	123 (71.9)	59 (74.6)	64 (70.6)	0.5	0.498	81 (76.4)	42 (65.6)	2.3	0.157
	Disagree	48 (28.1)	20 (25.4)	28 (29.4)			25 (23.6)	22 (34.4)		
I feel I have the skills required to be able to encourage older patients who smoke to make a quit attempt.	Agree	122 (71.7)	66 (83.5)	56 (61.5)	10.1	0.002	79 (75.2)	42 (65.6)	1.8	0.219
	Disagree	48 (28.3)	13 (16.5)	35 (38.5)			26 (24.8)	22 (34.4)		
I feel I have sufficient knowledge of nicotine replacement and other pharmacotherapies which can help older smokers to quit, and feel able to discuss this with patients.	Agree	115 (67.6)	60 (75.9)	55 (60.4)	4.6	0.034	74 (70.4)	41 (64.1)	0.7	0.400
	Disagree	55 (32.4)	19 (24.1)	36 (39.6)			31 (29.6)	23 (35.9)		
I am comfortable discussing my older patients smoking behaviours with them.	Agree	136 (80.4)	72 (92.3)	64 (70.3)	12.9	<0.001	87 (83.6)	49 (76.5)	1.3	0.312
	Disagree	33 (19.6)	6 (7.7)	27 (29.7)			17 (16.4)	15 (23.5)		
Smoking is one of the few pleasures older people have.	Agree	41 (24.1)	7 (8.9)	34 (37.4)	18.7	<0.001	20 (19.0)	21 (32.8)	4.1	0.043
	Disagree	129 (75.9)	72 (91.9)	57 (62.6)			85 (81.0)	43 (67.2)		
Giving up smoking is unlikely to provide older people with any benefit.	Agree	9 (5.2)	0 (0.0)	9 (9.8)	8.3	0.004	4 (3.8)	5 (7.8)	1.3	0.302
	Disagree	161 (94.8)	79 (100.0)	82 (90.2)			101 (96.2)	59 (92.2)		
I feel that it is the role of more specialist staff to actually support an older patient's cessation attempt.	Agree	51 (30.0)	24 (30.3)	27 (29.7)	0.10	1.000	26 (24.7)	25 (39.0)	3.9	0.058
	Disagree	119 (70.0)	55 (69.7)	64 (70.3)			79 (75.3)	39 (61.0)		

### Clinical practice with older smokers

Table 4 presents the frequency of respondents’ clinical practice relating to identifying and addressing tobacco dependence among older patients, by professional group and attendance at smoking related training. When compared to non-medical staff, medical staff were found to be significantly more likely to report regular proactive clinical practice in relation to addressing tobacco dependence among older people. However, when the non-medical staff group was sub-divided to allow for an exploration of differences, in comparison to healthcare assistants, nurses were significantly more likely to: make enquiries as to older patients smoking status [90.5 % vs 64.2 %, OR 3.51 (95 % CI 1.4–8.7), *p* = 0.021]; record older patients smoking status [93.2 % vs 64.2 %, OR 4.33, (95 % CI 1.8–10.3), *p* = 0.008; assess older smokers motivation to stop smoking [79.7 % vs 28.6 %, OR 6.30, (95 % CI 2.2–18.2), *p* = <0.001; provide older smokers with brief advice in relation to smoking [82.4 % vs 21.4 %, OR 9.78, (95 % CI 2.98–32.1), *p* = <0.001]; discuss the benefits of smoking cessation with older patients [75.7 % vs 7.1 %, OR 23.9, (95 % CI 3.3–174.2-), *p* = <0.001]; and supporting smoking cessation attempts including referral to stop smoking services [55.4 % vs 21.4 %, OR 3.67, (95 % CI 1.1–12.6), *p* = 0.039]. The overall median score summarising respondents’ clinical practice relating to treating tobacco dependence among older smokers was 12 out of a maximum of 18 (2–18). Nurses achieved a mean score of 12.8 (SD 3.6), while doctors and HCA’s obtained a mean score of 12.3 (SD 2.8) and 8.3 (SD 3.8) respectively.

**Table 4:** Frequency of respondents’ clinical practice to identify and address tobacco dependence among older patients

Statement		All responses *n* = (%)	Professional Group [*n* = (%)]				Training Attendance [*n* = (%)]			
			Medical	Non-medical	Statistic *X* ^2^ (df = 1)	Sig p=	Training	No Training	Statistic *X* ^2^ (df = 1)	Sig p=
How often do you ask older patients about their smoking status?	Always/often	154 (90.1)	76 (96.2)	78 (84.8)	6.19	0.019	98 (92.5)	55 (85.9)	1.88	0.170
	Rarely/never	17 (9.9)	3 (3.8)	14 (15.2)			8 (7.5)	9 (14.1)		
How often did you document an older smokers' smoking status in their clinical records?	Always/often	155 (90.1)	75 (94.9)	80 (86.9)	3.19	0.112	100 (94.3)	54 (84.3)	4.65	0.031
	Rarely/never	16 (9.9)	4 (5.1)	12 (13.1)			6 (5.7)	10 (15.7)		
How often did you assess older smokers' motivation/readiness to stop smoking?	Always/often	130 (75.9)	65 (82.3)	65 (70.7)	3.15	0.105	89 (83.9)	40 (62.5)	10.04	0.002
	Rarely/never	41 (24.1)	14 (17.7)	27 (29.3)			17 (16.1)	24 (37.5)		
How often did you provide older smokers with brief advice or encouragement to consider stopping smoking?	Always/often	133 (77.8)	67 (84.8)	66 (71.7)	4.20	0.040	86 (81.1)	46 (71.8)	1.97	0.185
	Rarely/never	38 (22.2)	12 (15.2)	26 (28.3)			20 (18.9)	18 (28.2)		
How often did you discuss the benefits of stopping smoking with the older smokers you have had contact with?	Always/often	123 (71.9)	63 (79.7)	60 (65.2)	4.44	0.041	84 (79.2)	38 (59.4)	7.78	0.008
	Rarely/never	48 (28.1)	16 (20.3)	32 (34.8)			22 (20.8)	26 (40.6)		
How often did you provide older smokers with support (including NRT or referral to local stop smoking services) to make a quit attempt?	Always/often	100 (59.1)	55 (71.4)	45 (48.9)	8.80	0.005	69 (65.7)	30 (47.6)	5.33	0.024
	Rarely/never	69 (40.9)	22 (28.6)	47 (51.1)			36 (34.3)	33 (52.4)		

Training was found to have a positive association with a number of the clinical practices assessed in this survey. Overall, those clinicians who had received smoking related training achieved a slightly higher mean score in relation to their practice of addressing smoking among older adults when compared to those who had not attended smoking related training [12.79 (SD 3.2) vs 11.13 (SD 3.6), respectively].

### Reported barriers to addressing tobacco dependence among older people in primary care

Thirty-one participants (18.1 %) cited time constraints as a barrier to addressing tobacco use among older patients. While only 6.5 % of clinicians stated that they did not believe it was part of their role to discuss smoking and smoking cessation with their older patients, significantly more non-medical staff were likely to adopt this view than medical staff (10.9 % vs 1.3 %, *p* = 0.012). Similarly, with less than five percent (4.8 %) of respondents believing it unfair to ask older people to quit, non-medical staff were found to be statistically more likely to believe this in contrast to medical staff (8.7 % vs 0.0 %, *p* = 0.008). Only 3.5 % (*n* = 6) of respondents believed that raising the subject of smoking would damage the patient-clinician relationship and 40 (23.4 %) respondents believed that it was not appropriate to broach the subject of smoking with older patients due to conditions such as dementia, terminal illness, and mental disorders.

### Logistic regression analysis

Professional group and training attendance variables shown to have a positive effect on attitudes and clinical practice at *p* = 0.05 level, were entered into a logistic regression model. Training attendance by non-medical staff was found to increase the odds of disagreeing with the statement “smoking is one of the few pleasures older people have” by 2.1 times (95 % CI 1.1-4.2, *p* = 0.045). Training increased the likelihood of respondents practice relating to discussing the benefits of smoking cessation with older patients by 2.3 times (95 % CI 1.1-4.9, *p* = 0.019) among medical staff and by 2.9 times for non-medical staff (95 % CI 1.4-5.6, *p* = 0.003). Finally, the odds of respondents providing older patients with smoking cessation support increased with training by 2.4 times for medical (95 % CI 1.2-4.7, *p* = 0.009) and by 3.0 times for non-medical staff (95 % CI 1.4-5.6, *p* = 0.002).

## Discussion

The findings of this study suggest that, while primary care clinicians have overall adopted proactive practices towards addressing tobacco dependence among older patients, opportunities to encourage and support cessation might be missed through lack of awareness of the impacts of smoking and the benefits of smoking cessation in later life specifically.

To our knowledge, this is the first survey to explore the treatment of tobacco dependence specifically relating to older adults among NHS primary care clinicians in England. While the response rate (40 %) of this study is a limitation, it is noteworthy that it compares favourably with response rates found in recent surveys conducted among primary care clinicians in the UK [17, 18]. The generalizability of results may further be limited by survey-typical reporting bias; however, due to the inclusion of 58 general practices and 4 specialist community teams in a large and demographically diverse English city, we believe that this survey constitutes a valuable contribution to the literature. Furthermore the gender characteristics and smoking prevalence of participants comparable to that reported to exist among the primary care workforce; for example a study conducted by Drennan and colleagues [19] found a female predominance (96 %) among nurses and healthcare assistants working in general practice settings with a smoking prevalence of 5 %, while smoking among GP’s has been reported to be 4 % [20]. While the number of healthcare assistants who completed the survey is lower in comparison to other staff groups, compares favourably with the reported 9.3 % of healthcare assistants working within primary care [21].

Considerable inconsistency was identified between clinician staff groups in their knowledge of tobacco dependence and its treatment in older adults. If tobacco use among older people is to be effectively addressed, knowledge in relation to its prevalence, impacts, and treatment is essential. However, in this survey the majority of respondents believed smoking prevalence within the general and elderly populations to be in excess of published statistics, and few reported an awareness of the number of life years lost to smoking related disease. Despite medical staff showing a greater awareness of the impact of smoking and smoking cessation specific to older people, gaps in knowledge were present across all staff groups. A lack of awareness relating to the interaction between smoking and medications used to treat conditions in old age is of particular clinical importance, since it may result in ineffective prescribing or in an exacerbation of chronic conditions: smoking can affect mean serum levels for drugs such as propranolol and theophylline, and is widely reported to decrease levels of anti-depressants, anti-psychotic, and opiate based medication; additionally, heavy smokers require one and a half times more insulin to control their diabetes than non-smokers [22].

In view of the important role clinicians in primary care play in smoking cessation, the overall positive attitudes towards addressing tobacco dependence among older patients are encouraging. However, while the majority of clinicians believed that it was within their remit of responsibility as a healthcare professional to assist older smokers to address their tobacco dependence and a significant number of respondent medical staff reported having sufficient knowledge and skills to support cessation, non-medical staff reported feeling less confident in their ability. As nurses and healthcare assistants play an important role in primary care, particularly in terms of health promotion, screening, and monitoring activity, it is concerning that as many as 38.5 % of non-medical staff reported lacking confidence in knowledge and skills required to be able to effectively encourage and support older patients in addressing their tobacco dependence.

The lack of association between training attendance and clinician attitudes and knowledge is remarkable. While, the survey did not assess the content or nature of smoking related training received by clinicians, it is likely that primary care staff would receive training in the promotion of smoking cessation as part of their pre-or post-registration education or professional development. To fully understand the impact of training on the attitudes and knowledge of clinicians further evidence is required on the type and content of training received by GP’s, nurses, and healthcare assistants.

Previous research indicates that older people experience a number of psychological, physical, and social barriers to making a quit attempt and in accessing smoking cessation services [23, 24]. In addition, clinicians have been shown to believe that long term smokers are less willing to give up or are less receptive to brief advice [13].

Our study found that just under half of all respondents were unaware of the barriers to smoking cessation faced by older patients, and nearly 30 % felt that it was the task of specialist staff to support older people in their cessation attempts. In addition, over one third of non-medical staff felt that “smoking is one of the few pleasures older people have”. These attitudes are likely to impact on the clinicians’ motivation to offer cessation support to older smokers, and the way they offer it. Furthermore, and similar to previous studies [25–27], clinicians reported a perceived lack of time during consultation with older patients to be able to provide smoking cessation advice and treatment. The benefits of quitting smoking in later life are well documented, as are the potential improvements to chronic, long-term and life-limiting conditions and patients’ quality of life. Despite this, our study found that nearly a quarter of respondents did not feel it was appropriate to discuss smoking cessation with patients with dementia, terminal illness, or mental disorder. Just over 3 % reported a reluctance to discuss patients’ smoking for fear of negative impacts on the therapeutic relationship, a finding that does not replicate the results of Coleman’s study [25], which found that many of the 42 GP’s interviewed cited that fear of harming the doctor-patient relationship was a barrier to discussing smoking with patients. While it is understandable that clinicians do not wish to add to patients health or social burdens or to stigmatise those who may be suffering from smoking related diseases, there is recent evidence to suggest that patients will accept life-style advice about behaviours associated with their illness [28]. Research conducted by Gritz et al. [29] highlighted the importance of clinical staff in promoting smoking cessation in cancer patients, suggesting that diagnosis and on-going clinical contacts provide opportunities for teachable moments and an improvement in condition or the effects of treatment [30].

## Conclusion

Despite encouraging attitudes towards addressing tobacco use among older adults generally, our results across all staff groups show that opportunities for assisting older patients to address their tobacco dependence might currently be missed. There is a need to develop clinician capacity, knowledge and awareness related to smoking cessation in older people specifically, and to build confidence in seizing teachable moments, even or especially among those older people with chronic or terminal illnesses.

## Additional file

Additional file 1:
**Primary care clinician survey.**

## Abbreviations

CCG: Clinical commissioning group
GP: General practice/general practitioner
HCA: Healthcare assistant
NHS: National health service
UK: United Kingdom
