# Supplementary material for: Treating tobacco dependence in older adults: a survey of primary care clinicians’ knowledge, attitudes, and practice
Source: BMC Fam Pract. 2015 Aug 6;16:97. doi: 10.1186/s12875-015-0317-7 (PMC4527299; doi:10.1186/s12875-015-0317-7)
Supplement: Additional file 1: — Primary care clinician survey. [file 12875_2015_317_MOESM1_ESM.docx]

**Developing a tailored smoking cessation support model for the elderly in primary care: A cross-sectional survey of clinician knowledge, attitudes, and practice**

| **ABOUT YOU** | | | | | | |  |
| --- | --- | --- | --- | --- | --- | --- | --- |
|  |  | |  | |  | |  |
| 1. | What is your current role? | |  | |  | |  |
|  | GP | |  | | | |  |
|  | GP Trainee | |  | | | |  |
|  | Practice Nurse | |  | | | |  |
|  | Specialist/Community Nurse | |  | | | |  |
|  | Student Nurse | |  | | | |  |
|  | Healthcare Assistant | |  | | | |  |
|  | Allied Health Professional | |  | | | |  |
|  | Other (please specify below) | |  | | | |  |
|  |  | | | |  | |  |
|  |  | |  | |  | |  |
| 2. | Are you? | |  | |  | |  |
|  | Male | |  | | | |  |
|  | Female | |  | | | |  |
|  |  | |  | |  | |  |
| 3. | What is your age? | |  | |  | |  |
|  | Under 20 years | |  | | | |  |
|  | 20-29 years | |  | | | |  |
|  | 30-39 years | |  | | | |  |
|  | 40-49 years | |  | | | |  |
|  | 50-59 years | |  | | | |  |
|  | 60-69 years | |  | | | |  |
|  |  | |  | |  | |  |
| 4. | Are you a current smoker? | |  | |  | |  |
|  | Yes | |  | | | |  |
|  | No | |  | | | |  |
|  |  | |  | |  | |  |
|  | If yes, how many cigarettes do you smoke each day? | |  | |  | |  |
|  | Less than 10 | |  | | | |  |
|  | 11-20 cigarettes | |  | | | |  |
|  | 21-30 cigarettes | |  | | | |  |
|  | More than 31 cigarettes | |  | | | |  |
|  |  | |  | |  | |  |
| 5. | Have you received any training in relation to smoking? | |  | |  | |  |
|  | Yes | |  | | | |  |
|  | No | |  | | | |  |
|  |  | |  | |  | |  |
| **KNOWLEDGE OF TOBACCO DEPENDENCE AND ITS TREATMENT IN OLDER PEOPLE** | | | | | | |  |
|  |  | | | |  | |  |
| Please draw on your current knowledge when answering the questions below. | | | | | | |  |
| 6. | Approximately what percentage of people in England currently smokes? | |  | |  | |  |
|  | 10% | |  | | | |  |
|  | 20% | |  | | | |  |
|  | 30% | |  | | | |  |
|  | 40% | |  | | | |  |
|  | Don’t know | |  | | | |  |
|  |  | |  | |  | |  |
| 7. | Approximately, what percentage of people over the age of 65 currently smokes? | | | |  | |  |
|  | 10% | |  | | | |  |
|  | 25% | |  | | | |  |
|  | 30% | |  | | | |  |
|  | 45% | |  | | | |  |
|  | Don’t know | |  | | | |  |
|  |  | |  | |  | |  |
| 8. | How many years on average, do people who smoke regularly and die from smoking related disease loose from their life expectancy compared with a non-smoker? | | | |  | |  |
|  | 5 years | |  | | | |  |
|  | 10 years | |  | | | |  |
|  | 15 years | |  | | | |  |
|  | 20 years | |  | | | |  |
|  | Don’t know | |  | | | |  |
|  |  | |  | |  | |  |
| 9. | Which of the following statements are true, in relation to older people who smoke? (select all that apply) | | | |  | |  |
|  | Smoking can reduce the effectiveness of certain medications that are prescribed for conditions which are common in later life (e.g. CHD, arthritis, hypertension). | |  | | | | |
|  |  | |  | |  | |  |
|  | Smoking can delay wound healing in older people. | |  | | | |  |
|  |  | |  | |  | |  |
|  | The risk of an older person having a heart attack or a stroke increases within 24 hours of stopping smoking. | |  | | | |  |
|  |  | |  | |  | |  |
|  | Smoking can speed cognitive decline in older people. | |  | | | |  |
|  |  | |  | |  | |  |
|  | Stopping smoking does not generally stop the progression of chronic obstructive pulmonary disease. | |  | | | |  |
|  |  | |  | |  | |  |
|  | Smoking can cause serious complications in older people with diabetes. | |  | | | |  |
|  |  | |  | |  | |  |
|  | Older smokers are less likely to want to quit smoking. | |  | | | |  |
|  |  | |  | |  | |  |
|  | Brief smoking cessation advice less effective than more intensive advice in helping older people to stop smoking. | |  | | | |  |
|  |  | |  | |  | |  |
|  |  | |  | |  | |  |
|  |  | |  | |  | |  |
| **THERAPEUTIC ATTITUDES REGARDING TOBACCO DEPENDENCE AMONG OLDER PEOPLE** | | | | | | |  |
|  | |  | |  | |  |  |
| Please indicate the extent of your agreement or disagreement with each of the following statements about working with older people who smoke. | | | | | | |  |
|  | |  | | | |  |  |
| 10. | | I feel it lies within my remit of responsibility as a health professional to assist older people who smoke to address their tobacco dependence. | |  | |  |  |
|  | | Strongly agree | |  | | |  |
|  | | Agree | |  | | |  |
|  | | Neither agree or disagree | |  | | |  |
|  | | Disagree | |  | | |  |
|  | | Strongly disagree | |  | | |  |
|  | |  | |  | |  |  |
| 11. | | I feel I have sufficient knowledge of the detrimental effects of smoking in later life to be able to discuss this effectively when I have contact with older patients who smoke | | | |  |  |
|  | | Strongly agree | |  | | |  |
|  | | Agree | |  | | |  |
|  | | Neither agree or disagree | |  | | |  |
|  | | Disagree | |  | | |  |
|  | | Strongly disagree | |  | | |  |
|  | |  | |  | |  |  |
| 12. | | I feel I have sufficient knowledge of the benefits of stopping smoking in later life to be able to discuss this effectively with older patients who smoke. | | | |  |  |
|  | | Strongly agree | |  | | |  |
|  | | Agree | |  | | |  |
|  | | Neither agree or disagree | |  | | |  |
|  | | Disagree | |  | | |  |
|  | | Strongly disagree | |  | | |  |
|  | |  | |  | |  |  |
| 13. | | I feel I have sufficient knowledge of the barriers that may prevent older people from attempting to stop smoking. | | | |  |  |
|  | | Strongly agree | |  | | |  |
|  | | Agree | |  | | |  |
|  | | Neither agree or disagree | |  | | |  |
|  | | Disagree | |  | | |  |
|  | | Strongly disagree | |  | | |  |
|  | |  | |  | |  |  |
| 14. | | I feel I have sufficient knowledge of nicotine addiction/withdrawal to be able to discuss this effectively with older patients who smoke. | |  | |  |  |
|  | | Strongly agree | |  | | |  |
|  | | Agree | |  | | |  |
|  | | Neither agree or disagree | |  | | |  |
|  | | Disagree | |  | | |  |
|  | | Strongly disagree | |  | | |  |
|  | |  | |  | |  |  |
| 15. | | I feel I have the skills required to be able to encourage older patients who smoke to make a quit attempt. | |  | |  |  |
|  | | Strongly agree | |  | | |  |
|  | | Agree | |  | | |  |
|  | | Neither agree or disagree | |  | | |  |
|  | | Disagree | |  | | |  |
|  | | Strongly disagree | |  | | |  |
|  | |  | |  | |  |  |
| 16. | | I feel I have sufficient knowledge of nicotine replacement and other pharmacotherapies which can help older smokers to quit, and feel able to discuss this with patients. | | | |  |  |
|  | | Strongly agree | |  | | |  |
|  | | Agree | |  | | |  |
|  | | Neither agree or disagree | |  | | |  |
|  | | Disagree | |  | | |  |
|  | | Strongly disagree | |  | | |  |
|  | |  | |  | |  |  |
| 17. | | I am comfortable discussing my older patients smoking behaviours with them. | | | |  |  |
|  | | Strongly agree | |  | | |  |
|  | | Agree | |  | | |  |
|  | | Neither agree or disagree | |  | | |  |
|  | | Disagree | |  | | |  |
|  | | Strongly disagree | |  | | |  |
|  | |  | |  | |  |  |
| 18. | | Smoking is one of the few pleasures older people have. | |  | |  |  |
|  | | Strongly agree | |  | | |  |
|  | | Agree | |  | | |  |
|  | | Neither agree or disagree | |  | | |  |
|  | | Disagree | |  | | |  |
|  | | Strongly disagree | |  | | |  |
|  | |  | |  | |  |  |
| 19. | | Giving up smoking is unlikely to provide older people with any benefit. | |  | |  |  |
|  | | Strongly agree | |  | | |  |
|  | | Agree | |  | | |  |
|  | | Neither agree or disagree | |  | | |  |
|  | | Disagree | |  | | |  |
|  | | Strongly disagree | |  | | |  |
|  | |  | |  | |  |  |
| 20. | | I feel that it is the role of more specialist staff to actually support an older patient's cessation attempt. | |  | |  |  |
|  | | Strongly agree | |  | | |  |
|  | | Agree | |  | | |  |
|  | | Neither agree or disagree | |  | | |  |
|  | | Disagree | |  | | |  |
|  | | Strongly disagree | |  | | |  |
|  | |  | |  | | |  |
| 21. | | I am interested to see what can be done to encourage older people to stop smoking. | |  | |  |  |
|  | | Strongly agree | |  | | |  |
|  | | Agree | |  | | |  |
|  | | Neither agree or disagree | |  | | |  |
|  | | Disagree | |  | | |  |
|  | | Strongly disagree | |  | | |  |
|  | |  | |  | |  |  |
| **CLINICAL PRACTICE WITH OLDER SMOKERS** | | | | | | |  |
|  | |  | |  | |  |  |
| 22. | | How often do you ask older patients about their smoking status (whether they are a smoker/former smoker, or how many cigarettes they smoke on a daily basis)? | | | |  |  |
|  | | Always | |  | | |  |
|  | | Often | |  | | |  |
|  | | Rarely | |  | | |  |
|  | | Never | |  | | |  |
|  | |  | |  | |  |  |
| 23. | | How often did you document an older smokers' smoking status in their clinical records? | | | |  |  |
|  | | Always | |  | | |  |
|  | | Often | |  | | |  |
|  | | Rarely | |  | | |  |
|  | | Never | |  | | |  |
|  | |  | |  | |  |  |
| 24. | | How often did you assess older smokers' motivation/readiness to stop smoking? | |  | |  |  |
|  | | Always | |  | | |  |
|  | | Often | |  | | |  |
|  | | Rarely | |  | | |  |
|  | | Never | |  | | |  |
|  | |  | |  | |  |  |
| 25. | | How often did you provide older smokers with brief advice or encouragement to consider stopping smoking? | |  | |  |  |
|  | | Always | |  | | |  |
|  | | Often | |  | | |  |
|  | | Rarely | |  | | |  |
|  | | Never | |  | | |  |
|  | |  | |  | |  |  |
| 26. | | How often did you discuss the benefits of stopping smoking with the older smokers you have had contact with? | |  | |  |  |
|  | | Always | |  | | |  |
|  | | Often | |  | | |  |
|  | | Rarely | |  | | |  |
|  | | Never | |  | | |  |
|  | |  | |  | |  |  |
| 27. | | How often did you provide older smokers with support (including NRT or referral to local stop smoking services) to make a quit attempt? | | | |  |  |
|  | | Always | |  | | |  |
|  | | Often | |  | | |  |
|  | | Rarely | |  | | |  |
|  | | Never | |  | | |  |
|  | |  | |  | |  |  |
| 28. | | If you answered 'rarely' or 'never' to any of the above questions, it would be useful if you could provide some information on factors which have influenced your practice with the older people who smoke that you have had contact with. (select all that apply) | | | | |  |
|  | |  | |  | |  |  |
|  | | I do not generally have time to discuss smoking/smoking cessation. | |  | | |  |
|  | | I do not think that it is part of my role to discuss smoking/smoking cessation. | |  | | |  |
|  | | I think it is unfair to ask older people to stop smoking. | |  | | |  |
|  | | I am concerned that mentioning stopping smoking would damage the relationship I have with the older smokers I have contact with. | |  | | |  |
|  | | A number of the older people I have contact with have dementia and I do not feel it would be appropriate to broach the subject of stopping smoking in these circumstances. | |  | | |  |
|  | | A number of the older people I have contact with have a terminal illness and I do not feel it would be appropriate to broach the subject of stopping smoking in these circumstances. | |  | | |  |
|  | | A number of the older people I have contact with have a mental health problem and I do not feel it would be appropriate to broach the subject of stopping smoking in these circumstances. | |  | | |  |
|  | |  | |  | |  |  |
| 29. | | We would be very interested in hearing about your clinical experiences with older smokers. If you would like to share your experiences, comments, or suggestions regarding smoking or smoking cessation among older people within your primary care activities, please use the box below. | | | |  |  |
|  | |  | | | |  |  |
|  | |  |  |  |  |  |  |
|  | |  |  |  |  |  |  |
|  | |  |  |  |  |  |  |
|  | |  |  |  |  |  |  |
|  | |  |  |  |  |  |  |
|  | |  |  |  |  |  |  |
|  | |  |  |  |  |  |  |
|  | |  |  |  |  |  |  |
|  | |  | |  | |  |  |

**Thank you for your help!**
